# Lemierre's Syndrome: Recognising a Typical Presentation of a Rare Condition

**DOI:** 10.1155/2015/797415

**Published:** 2015-01-27

**Authors:** James A. Coultas, Neena Bodasing, Paul Horrocks, Anthony Cadwgan

**Affiliations:** ^1^Keele University Medical School, Keele University, Staffordshire ST5 5BG, UK; ^2^Royal Stoke University Hospital, Newcastle Road, Stoke-on-Trent, Staffordshire ST4 6QG, UK

## Abstract

Lemierre's syndrome is a rare complication following an acute oropharyngeal infection. The aetiological agent is typically anaerobic bacteria of the genus* Fusobacterium*. The syndrome is characterised by a primary oropharyngeal infection followed by metastatic spread and suppurative thrombophlebitis of the internal jugular vein. If left untreated, Lemierre's syndrome carries a mortality rate of over 90%. Whilst relatively common in the preantibiotic era, the number of cases of Lemierre's syndrome subsequently declined with the introduction of antibiotics. With the increase of antibiotic resistance and a greater reluctance to prescribe antibiotics for minor conditions such as tonsillitis, there are now concerns developing about the reemergence of the condition. This increasing prevalence in the face of an unfamiliarity of clinicians with the classical features of this “forgotten disease” may result in the misdiagnosis or delay in diagnosis of this potentially fatal illness. This case report illustrates the delay in diagnosis of probable Lemierre's syndrome in a 17-year-old female, its diagnosis, and successful treatment which included the use of anticoagulation therapy. Whilst there was a positive outcome, the case highlights the need for a suspicion of this rare condition when presented with distinctive signs and symptoms.

## 1. Introduction

Lemierre's syndrome is caused by bacteria of the genus* Fusobacterium*, most commonly* F. necrophorum*, but occasionally* F. nucleatum*, *F. mortiferum*, and* F. varium *[1–4]. These are gram negative, nonmotile, nonsporulating, pleomorphic, and anaerobic bacilli with filamentous ends, which are normal flora of the gastrointestinal tract, oropharynx, and female urogenital tract [1, 5]. The pathogenesis of the disease remains unknown, although there are several theories [6]. As* F. necrophorum* is present in the oropharynx of healthy patients, the pathogenesis of Lemierre's syndrome must involve factors that facilitate the invasion through the mucosa [7]. One theory suggests that the integrity of the oral mucosa is altered through the presence of a viral or bacterial pharyngitis, and it is currently known that around one third of patients have a polymicrobial bacteraemia, with streptococcal infections being the most common [6, 8]. There are also reports of Lemierre's syndrome following infection with Epstein-Barr virus, again supporting the theory of a concomitant infection facilitating a fusobacterium invasion [9]. Once the invasion of the pharyngeal mucosa has occurred, the proximity of the internal jugular vein allows for further spread of the fusobacteria from the peritonsillar space into this vessel [1, 2, 4]. Platelet aggregation and subsequent thrombus formation in the internal jugular vein are a direct consequence of this bacteraemia, and this also acts a source of metastatic septic emboli [1, 4, 10]. In a few cases, thrombophlebitis has been identified in some of the branches of the external jugular vein, in addition to the internal jugular vein [11].

Lemierre's syndrome was common before the discovery of antibiotics [9]. With increased use of penicillin for bacterial throat infections from the 1960s, the number of reports of Lemierre's syndrome dropped at this time [7, 12–14]. However, there is evidence to suggest that the incidence of this disease is rising [2, 15, 16]. Despite such reports, Lemierre's syndrome remains a rare condition, with one retrospective study from Denmark estimating an incidence of around 1 case per million [8]. This case report highlights the challenge in the timely diagnosis of Lemierre's syndrome.

## 2. Case Report

A 17-year-old female presented at the emergency department following a 7-day history of worsening sore throat, fever, headache, and vomiting and a 4-day history of developing neck pain. Her illness initially started with fever, headache, and sore throat with subsequent loss of appetite. The headache was reported to fluctuate in severity and was described more like a migraine, although it was relieved slightly by paracetamol. The sore throat was severe enough to affect her intake of food, as she was unable to swallow solids, but liquids were unaffected. The patient reported vomiting following the onset of the illness, although this only initially occurred after eating and there was no blood or bile present. The vomiting subsided in the following days due to decreased intake of food. However, one more episode of vomiting occurred the day before admission which was reported to be bilious. The patient had previously been seen by her general practitioner and at the emergency department on two previous occasions (four and three days before admission). She was discharged on all occasions with the diagnosis of a self-limiting viral illness. Subsequently, she developed a left-sided neck pain, radiating down the lateral margin of her neck (ranked as 6/10 for severity) and was admitted to the emergency department via an ambulance.

**Figure 1:**
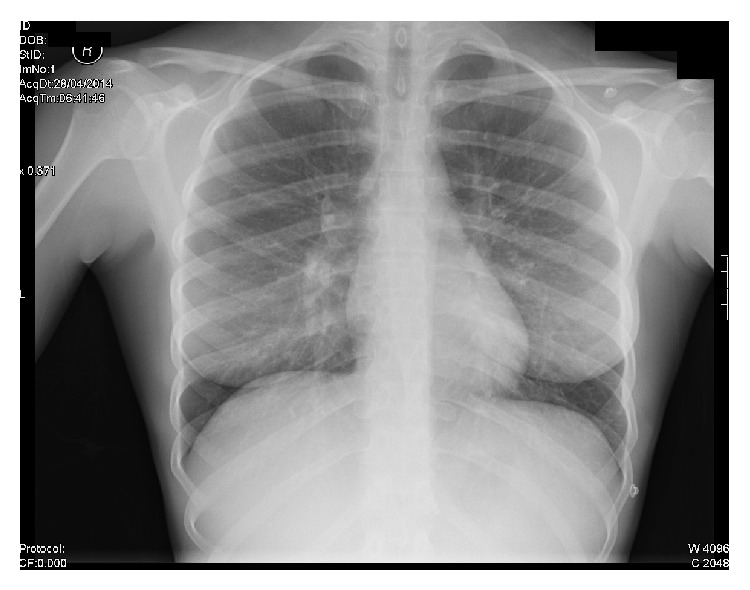
Erect chest radiograph on admission.

**Figure 2:**
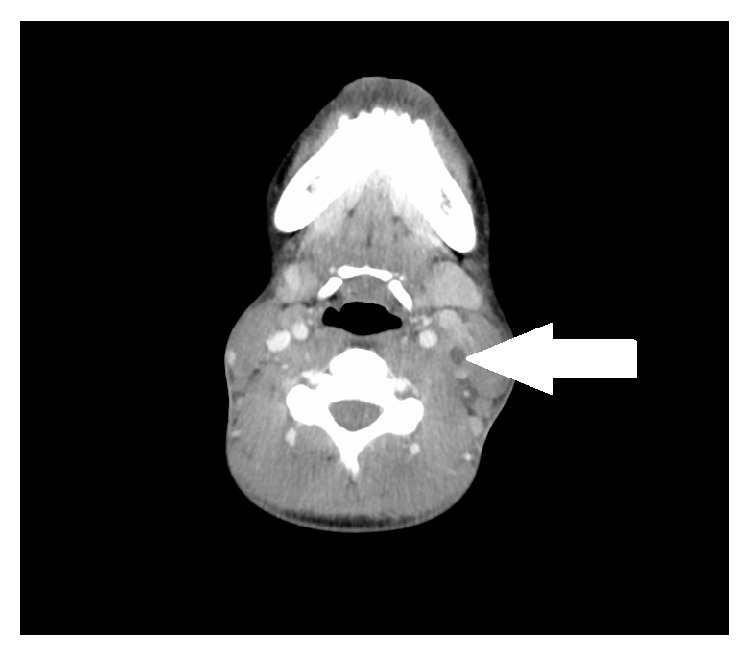
Contrast CT scan of the neck showing a thrombus in the left jugular vein (arrow).

**Figure 3:**
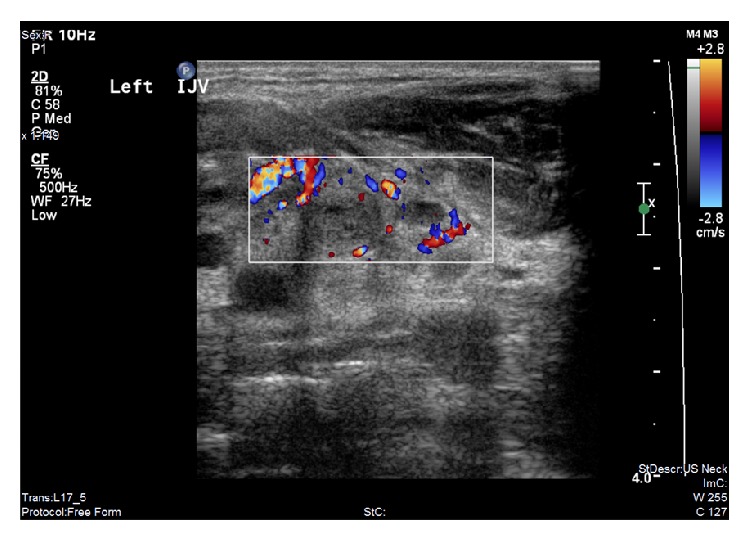
Doppler ultrasound of the neck showing the thrombus in the internal jugular vein.

On admission, there was swelling of the left neck and pain on movement, although there was no photophobia, and on examination she had a negative Kernig's and Brudzinski sign. Her temperature was 39.9°C, respiratory rate was 18 breaths/min, blood pressure was 105/44 mmHg, and heart rate was 137 bpm. The patient's blood results showed evidence of liver dysfunction with decreased albumin of 28 g/L, raised alkaline phosphatase of 178 U/L, raised alanine transaminase of 50 U/L, raised bilirubin of 28 *μ*mol/L, and raised gamma-glutamyl transferase of 200 U/L. They also showed a raised C-reactive protein (CRP) of 241 mg/L, reduced haemoglobin of 105 g/L, reduced red blood cell count of 3.54 × 10^12^/L, and a reduced haematocrit of 0.32. The absolute neutrophil count was raised at 8.50 × 10^9^/L; however, the absolute lymphocyte count was reduced at 0.40 × 10^9^/L, whilst the white cell count was within the normal range at 9.2 × 10^9^/L. Platelet count was also normal at 212 × 10^9^/L. Chest X-ray, computerised tomography (CT) neck, CT pulmonary angiogram, and ultrasound of the neck were performed. The chest X-ray (Figure 1) showed prominent vascular appearances to the hilar contours, but no obvious paratracheal hilar lymphadenopathy. There was also a slight increase in perihilar bronchovascular markings. A second chest X-ray was performed 4 days later to look for an infective focus in the chest; none was found (not shown). The CT scan of the neck with contrast showed a thrombus within the left jugular vein (Figure 2). The thrombus did not extend into the venous sinuses in the brain or into the mediastinum. The CT pulmonary angiogram showed no evidence of pulmonary embolism or lung pathology. Ultrasound of the neck (Figure 3) showed left neck soft tissue swelling, reactive lymphadenopathy, and left internal jugular vein thrombosis. At this point a diagnosis of Lemierre's syndrome was made. Blood cultures were taken on admission and were returned negative five days later. A second set of blood cultures were repeated at this time, with these also returned as negative. However, it is noteworthy that these blood cultures were taken after the patient had received antibiotic therapy in the community as well as after admission to the emergency department.

The patient was given benzylpenicillin (2.4 g IM) and paracetamol (1 g PO) by the ambulance crew prior to admission, before being commenced on intravenous co-amoxiclav (1.2 g tds) and oral clarithromycin (500 mg bd) as well as therapeutic low molecular weight heparin (dalteparin 10000 units S/C od). Following the diagnosis of Lemierre's syndrome one day after admission, the antibiotic regimen was switched to IV clindamycin (1.2 g tds) and IV metronidazole (500 mg tds), whilst the IV co-amoxiclav was continued. These were changed to oral amoxicillin (1 g tds) and oral metronidazole (400 mg tds) 9 days after admission. She was also started on warfarin (5 mg PO od), 9 days after admission and continued receiving therapeutic dalteparin as from admission.

At discharge, 11 days following admission on the Infectious Diseases ward, her CRP levels had fallen to 6.4 mg/L, and the liver enzyme results had improved, although were not yet within the normal ranges. The patient was asymptomatic on discharge. Her course of antibiotics ended 11 days after discharge and she continued to take warfarin for 3 months, subject to the advice of the haematology department. The patient attended a follow-up outpatient appointment eight weeks later. Her inflammatory markers and liver enzymes had fallen to within the normal range and there was no fever, sore throat, or neck swelling.

## 3. Discussion

The apparent increasing prevalence of Lemierre's syndrome combined with the unfamiliarity of clinicians with its classical presenting features often initially results in misdiagnosis of an oropharyngeal infection. This case illustrates this scenario well, with the patient admitted to the emergency department via the ambulance service seven days after the onset of the initial oropharyngeal infection, with three separate diagnoses of self-limiting viral illness in this time. Following a more acute presentation and appropriate radiological imaging investigations, Lemierre's syndrome was diagnosed here and appropriately treated. Whilst rare, the presenting history of a recurrent sore throat with developing neck pain in an otherwise healthy adolescent/young adult should lead to a high index of suspicion of Lemierre's syndrome so these cases can be diagnosed and early treatment initiated.

A review of the typical presentation of Lemierre's syndrome highlights some of the classical signs and symptoms described in this case presentation. The illness typically begins with a fever reaching 39–41°C, the first sign of septicaemia, which may or may not be accompanied by rigors [17]. The septicaemia is most commonly preceded by a sore throat which usually occurs 4-5 days before all other symptoms, but in some cases has been up to 12 days before [7]. The presentation of the sore throat varies, with many showing a normal appearance of the oropharynx [17]. However, in some cases, a severe exudative tonsillitis accompanied by peritonsillar abscess has been documented and may be severe enough to cause dysphagia [17]. Neck pain and stiffness are commonly described, and bilateral or unilateral cervical lymphadenopathy may be present, commonly in the anterior triangle. Patients may also exhibit an induration of the internal jugular vein, slightly inferior to the sternocleidomastoid muscle's anterior border [2, 4]. The lungs are the most common site for metastasis, and, in 85% of patients, septic emboli from the internal jugular vein metastasize through the pulmonary arteries resulting in pulmonary effusions, abscesses, and empyema [8]. Other pulmonary manifestations include pneumatoceles, pneumothorax, and acute respiratory distress syndrome, reported in around 10% of patients [18, 19]. Lemierre himself described a triad of pleuritic chest pain, dyspnoea, and haemoptysis and the presence of localised crackles and a pleural rub on auscultation [17, 20]. Chest radiographs frequently show multiple nodular infiltrates throughout both lungs, although it is not unusual for radiographs to be normal as reported here [12]. A metastatic infection found in Lemierre's syndrome can also manifest as septic arthritis, osteomyelitis, meningitis, pericarditis, and hepatic abscesses [18]. Septic arthritis has been reported to occur in 13–27% of cases, typically affecting the hip joint, whereas osteomyelitis only affects around 3% of patients [17]. Liver involvement often results in hepatomegaly and jaundice, and abdominal pain is not uncommon [18]. In such cases, and as shown here, liver function tests reveal raised liver enzymes and a low grade hyperbilirubinemia [18]. Patient's also typically display a neutrophil leukocytosis and an elevated CRP count [2, 17].

Chest radiograph has been demonstrated by Karkos et al. to be the most frequently ordered first line investigation, ordered in 92% of Lemierre's syndrome patients [21]. For patients with metastatic septic arthritis, arthrocentesis is performed, and the aspirated fluid has been reported to have a classical foul odour, but it is otherwise similar to other causes of septic arthritis [13]. For visualisation of the internal jugular vein, ultrasonography is often the first choice as it is relatively cheap, although it may miss thrombi with a low echogenicity and is less accurate at imaging inferior to the clavicle [22]. Contrast enhanced CT is more specific than ultrasonography and is often used for a definitive diagnosis [4]. MR imaging has been used in some cases where CT scanning has failed to detect a thrombus; however, this should not be used routinely as it is much more expensive [23]. Ultimately, detection and growth of a* Fusobacterium *spp. from anaerobic blood culture will provide the diagnosis [1, 6]. However, culturing may take up to 7 days, in which time any antibiotic treatment may decrease the likelihood of being able to grow the organism as was the case here [6, 17].

Various antibiotics have been proven to have* in vivo* activity against Fusobacteria, including lincomycin, clindamycin, minocycline, metronidazole, and less effectively penicillin and carbenicillin [5]. However, certain strains of* F. necrophorum* have reported resistance to penicillin due to beta-lactamase production [24]. Whilst there is no consensus on the antibiotic regimen; the use of a beta-lactam agent, such as penicillin, and metronidazole, for a period of a few weeks is recommended [25] and was used here. Intravenous antibiotics are preferred to oral regimens [26].

The patient was anticoagulated with low molecular weight heparin (dalteparin) after discussion with colleagues in haematology. She was subsequently commenced on warfarin for a period of 12 weeks of anticoagulation. The rare incidence of this syndrome results in a paucity of data for a systematic evaluation of the evidence base regarding the relative benefits and risks of anticoagulation, although reviews of case reports have been done [27–29]. Arguments against anticoagulation consider both the inherent risks of this therapy as well as the potential for metastatic spread of a septic emboli, noting that resolution of the internal jugular vein thrombosis typically occurs without anticoagulation. These risks are balanced against benefits that suggest that there is an increased resolution of the thrombus as well as penetration of antibiotics into the septic emboli. Here, the anticoagulation therapy was used to prevent embolization or extension of the thrombus. The INR was monitored closely due to the interaction of warfarin with metronidazole.

In conclusion, Lemierre's syndrome is a rare condition affecting primarily the young and should be suspected in a previously healthy young person who develops oropharyngeal infection and then exhibits signs and symptoms of internal jugular vein thrombophlebitis with or without sepsis. Blood cultures, chest radiographs, and contrast enhanced CT scanning should be definitive enough to provide a diagnosis. In this individual, the diagnosis was consistent with this syndrome, but confirmation and subspeciation of the infective aetiological agent were elusive due to prior antimicrobial therapy.
